# The Role of Advanced Practice Nurses in the Care of Multimorbid and Complex Chronically Ill Young and Middle-Aged Adults in Hospital Settings—Perspectives on Experience of APNs: A Qualitative Study

**DOI:** 10.3390/healthcare14121779

**Published:** 2026-06-19

**Authors:** Gabriele Bales, Birgit Schönfelder, Reto W. Kressig, Hanna Mayer

**Affiliations:** 1 Department of Nursing Science, Faculty of Social Sciences, University of Vienna, 1080 Vienna, Austria; 2 Department of Geriatric Medicine, Universitäre Altersmedizin Felix Platter, 4055 Basel, Switzerland; 3 Bachelor Nursing Programme, Faculty of Health, University of Applied Sciences Wiener Neustadt, 2700 Wiener Neustadt, Austria; 4 Faculty of Medicine, University of Basel, 4056 Basel, Switzerland; reto-w.kressig@unibas.ch; 5 Department of Nursing Science with Focus on Person-Centred Care Research, Faculty of Health Sciences, Karl Landsteiner University, 3500 Krems, Austria; hanna.mayer@kl.ac.at

**Keywords:** multimorbidity, complex chronic conditions, advanced practice nurses, young and middle-aged adults

## Abstract

**Highlights:**

**What are the main findings?**

- The perspectives of Advanced Practice Nurses (APNs) show that young and middle-aged adults with multimorbid and complex chronic illnesses require complex, long-term care that extends beyond the hospital setting, comprising various components that include person-centered care, transitional care, and continuity of care.
- APNs possess the skills and competencies—direct clinical practice, guidance and coaching, collaboration, and psychosocial support—to provide the necessary care for this patient group.

**What are the implications of the main findings?**

- A person-centered perspective is needed in continuous care; this perspective reflects an evolution from a disease-specific biomedical approach to a holistic, biopsychosocial understanding of the health and care needs of this patient group. This understanding must systematically consider and integrate the actual individual needs, personal experiences, and values of this patient group.
- This requires the implementation of projects that support the role of APNs in the clinical setting for the complex and comprehensive care of younger and middle-aged adults with multimorbid and complex chronic diseases, thus enabling this patient population to receive the care they need.

**Abstract:**

**Background/Objectives:** The rising prevalence of multimorbid and complex chronically ill young and middle-aged adults necessitates the implementation of innovative care models and the creation of roles that can meet the complex healthcare needs of this patient group. Advanced Practice Nurses (APNs) can play a crucial role in the care of multimorbid and complex chronically ill young and middle-aged adults in APN-led clinics; however, in Switzerland, these roles are still evolving. The aim of this study was to explore APNs’ perspectives on the planned development of their roles in an APN-led clinic. **Methods:** To gain insights into the experiences of APNs in caring for this patient group, a qualitative study design was chosen. Data were collected through interviews with APNs from Switzerland, the USA, and Canada. In total, 19 APNs (12 from Switzerland and 7 from the United States and Canada) participated in the study. The data were collected through semi-structured online interviews. These data were analyzed using reflective thematic analysis in accordance with the approach presented by Braun and Clarke. **Results:** The analysis identified 10 themes that describe the competencies, components, and framework conditions required for the work of APNs in an APN-led clinic for multimorbid and complex chronically ill young and middle-aged adults within the Swiss clinical context. Required competencies include direct clinical practice, guidance and coaching, collaboration, and psychosocial support. Essential components include person-centered care, transitional care, and continuity of care. Key framework conditions include regulations of the legal and regulatory framework and eligibility for reimbursement of services, resources, and extended competencies and scope of practice. **Conclusions:** The perspectives of the APNs involved in this study show that multimorbid and complexly chronically ill young and middle-aged adults require complex and long-term care that extends beyond the hospital setting. The findings of this study show that Swiss APNs may be well positioned to contribute to this role.

## 1. Introduction

The global prevalence of multimorbidity and complex chronic conditions is increasing, affecting not only older populations but also young and middle-aged adults (18–64 years) [1,2,3,4,5,6]. International studies suggest that a substantial proportion of this age group is already affected, with estimates indicating that up to 50% of young and middle-aged adults are affected by such conditions [7,8]. Emerging evidence further indicates that multimorbidity can already be present in early adulthood, with chronic conditions beginning to accumulate in individuals in their twenties [9]. Longitudinal studies demonstrate that multimorbidity in adults aged 18–65 years is characterized by distinct and evolving disease patterns over time, with cardiovascular and metabolic clusters playing a central role in morbidity and mortality risk [4,5]. Overall, these findings highlight that multimorbidity is not restricted to older age groups; rather, it represents a heterogeneous, life-course phenomenon affecting multiple age strata [3]. In Switzerland, a similar pattern can be observed, where multimorbidity is not limited to older populations, instead becoming relevant in middle adulthood. Data from the Swiss Health Observatory indicate that approximately 22% of the population aged 50 years and older are affected by such multimorbid conditions [10]; more than 40% of individuals over 55 years of age live with two or more chronic conditions. These include cardiovascular, rheumatic, pulmonary, and metabolic diseases (such as diabetes mellitus), as well as oncological and neurological conditions [11]. These disease groups represent some of the most resource- and cost-intensive burdens faced by the Swiss healthcare system, and the number of affected individuals is expected to increase further in the coming decades [12,13].

Adults with multimorbidity experience substantial impairments in their physical, psychosocial, and social functioning, including reduced health-related quality of life, functional limitations, and increased mortality risk [6,14,15,16,17,18,19]. Multimorbidity is also associated with a higher psychosocial burden, particularly when somatic and mental health conditions coexist, and with reduced capacity to manage treatment-related demands [14,17,20,21,22,23]. These combined burdens translate into increased healthcare utilization, higher rates of hospital admissions and readmissions, longer hospital stays, and more frequent instances of outpatient and primary care contact [21,24,25,26]. Importantly, multimorbidity is also linked to significant disruptions in work participation, productivity, and social functioning, particularly among working-age adults, highlighting that its impact extends well beyond older populations [6,27,28,29,30]. At the same time, affected individuals often experience fragmented care pathways, insufficient coordination between care providers, and treatment regimens that are not adequately adapted to multimorbidity, resulting in conflicting treatment recommendations and a lack of care coordination [15,22,31,32]. Overall, these findings highlight that multimorbidity is not only a condition that is defined by the coexistence of multiple diseases; it is also a complex and heterogeneous phenomenon characterized by distinct disease patterns and trajectories across different age groups. Emerging evidence further indicates that multimorbidity patterns vary over the life course, suggesting that younger and middle-aged adults may present with different clinical profiles and care needs compared to older populations [3]. This is further supported by longitudinal evidence demonstrating distinct multimorbidity trajectories across adulthood, with early accumulation of chronic conditions shaping long-term risk profiles [9]. Multimorbidity therefore challenges traditional single-disease-oriented healthcare models, as current services often fail to adequately address the complexity and interdependence of conditions. This underscores the need for holistic, person-centered, and coordinated models of care that ensure continuity across healthcare settings, particularly during transitions from hospital to home [33].

In response, integrated care approaches have been proposed as a key strategy to better manage complex chronic conditions, supporting coordination, self-management, and care that are tailored to individual needs, preferences, and capabilities [34,35,36,37]. Transitional care is an essential component of integrated care, ensuring the continuity of care during vulnerable transitions in the care trajectory and contributing to the improved long-term management of chronic conditions and the prevention of adverse outcomes [38]. At the core of many integrated care models are Advanced Practice Nurses (APNs), who play a central role in delivering, coordinating, and leading holistic, person-centered care; they ensure the continuity of care and facilitate seamless transitions from hospital to home [36,39,40,41,42]. According to the International Council of Nurses (ICN), an APN “is a generalist or specialized nurse who has acquired, through additional graduate education (minimum of a master’s degree), the expert knowledge base, complex decision-making skills and clinical competencies for Advanced Nursing Practice, the characteristics of which are shaped by the context in which they are credentialed to practice” (adapted from ICN, 2008) [43] (p. 1). The two most commonly identified APNs are nurse practitioners (NPs) and clinical nurse specialists (CNSs) [44]. CNSs primarily focus on improving the quality of care and patient outcomes through leadership, ensuring the proper education of staff nurses, and managing system-level interventions; NPs primarily engage in direct clinical practice, including advanced assessment, diagnosis, and treatment, and they often hold roles that overlap with those of physicians [45].

Despite increasing evidence on multimorbidity across the life course, APN-led models of care have predominantly been developed and implemented for older adult and geriatric populations. A scoping review by Bales et al. (2025) [46] identified only a very limited number of studies examining APN-led models of care for young and middle-aged adults with multimorbidity and/or complex chronic conditions in hospital and transitional care settings. Across the included studies, the evidence was scarce and heterogeneous, indicating a clear underrepresentation of this patient population in both integrated care and APN-related research. This highlights a substantial gap in the evidence base regarding the application of APN-led models within hospital-to-home transitional care pathways for younger and middle-aged adults [46]. In contrast to countries such as the United States and Canada, the development of APN roles in German-speaking countries, including Switzerland, is still in its early stages [47,48]. The development, implementation, and evaluation of these roles require the consideration of various perspectives, particularly those of APNs themselves [49,50]. Key aspects such as role clarity, role boundaries, and potential barriers and facilitators need to be systematically addressed to guarantee that APNs’ roles align with the values of nursing [49]. Currently, there are no APN roles within APN-led clinics in hospital settings in Switzerland that are specifically tailored to the care of young and middle-aged adults with multimorbid and complex chronic conditions. To begin addressing this evidence and practice gap, and to inform the development of such a model of care within the Swiss healthcare system, the aim of this study is to explore the perspectives of APNs in the context of the planned development of an APN role in an APN-led clinic. Therefore, the research focused on exploring competencies, components, and framework conditions from the perspectives of APNs. The research question to be investigated was the following: From the perspective of APNs, what competencies, components, and framework conditions are necessary for the development of this APN role concept?

## 2. Materials and Methods

### 2.1. Design

In order to gain an insight into the experiences of APNs caring for young and middle-aged adults with multimorbid and complex chronic conditions in a hospital setting, we chose a qualitative design in line with Braun and Clarke’s (2022) reflexive thematic analysis [51]. The data were collected in face-to-face interviews with APNs from the USA, Canada, and Switzerland. The reporting of this study is in line with the Standards for Reporting Qualitative Research (SRQR) [52].

### 2.2. Participants

Switzerland has APNs who provide care to younger and middle-aged adults with multimorbid and complex chronic illnesses in similar settings, but not in APN-led clinics within hospitals. Therefore, additional APNs from the USA and Canada were recruited as participants, as they work within such settings and already possess years of experience in APN roles within APN-led clinics providing care for younger and middle-aged individuals with multimorbid and complex chronic illnesses. Their expertise should therefore be utilized in developing an APN role for the care of this patient population. Inclusion criteria for participants in Switzerland were as follows: registered nurses in Switzerland who speak or understand German, hold a Master of Science in Nursing, and provide care in a hospital setting to young and middle-aged adult patients with multimorbidity and complex chronic conditions. In addition, participants were required to have been working in an APN role for at least three years. Inclusion criteria for participants in the United States and Canada were as follows: were registered nurses who hold a Master of Science in Nursing and provide care in a hospital setting to young and middle-aged adult patients with multimorbidity and complex chronic conditions. In addition, participants were required to have been working in an APN role for at least three years. Exclusion criteria for APNs in Switzerland, the United States, and Canada included the following: not being a registered nurse, not holding a Master of Science in Nursing, not working in a hospital setting with the defined patient population, or having less than three years of experience in an APN role. The recruitment of APNs was guided by both purposive and snowball sampling, as these complementary strategies were considered to be appropriate for meeting the study aims and addressing the challenges of accessing the target population. Purposive sampling was applied first to ensure the inclusion of APNs with specific experience in APN-led models of care, thereby securing a high level of relevance to the research question. Snowball sampling was subsequently used to extend recruitment through professional and personal networks, enabling access to additional eligible participants who might otherwise have been difficult to identify [53].

Interview participants from the USA and Canada were recruited using two different recruitment strategies: Firstly, APNs working in APN-led clinics internationally were contacted; these were identified on the basis of the literature review of international APN-led clinics for multimorbid and complex chronically ill young and middle-aged adults internationally [46,54]. These potential participants were invited via email to interview. Secondly, additional APNs within APN-led clinics in clinical settings were identified using a snowball sampling method via personal contacts. We also contacted these potential participants via email. We asked a total of nine APNs to participate in these interviews; of these, five from the USA and two from Canada expressed interest in participating. In Switzerland, recruitment was initiated with a call for study participation disseminated through SwissAPN. In a second step, participants from Switzerland were identified through snowball sampling (e.g., personal contacts) so that we could reach APNs working in different hospitals within the field of multimorbid and complex chronic conditions. We contacted potential participants by email. We asked 16 APNs to participate in these interviews; of these, 12 expressed an interest. Participants from both the USA/Canada and Switzerland were required to have at least three years of experience working as APNs in the care of multimorbid and complex chronically ill adults of younger and middle age. The reasons prospective participants declined to take part, regardless of origin, included a lack of interest or a lack of time.

### 2.3. Data Collection

Between August 2023 and November 2023, the first author—who has many years of expertise as an APN and experience conducting qualitative interviews—conducted interviews with 7 APNs from the USA and Canada (5 in the United States; 2 in Canada). The first author brought extensive professional expertise to the research context and was aware of the potential influence of prior assumptions throughout the research process, while actively striving to minimize this influence as much as possible. The interviews lasted between 28 and 90 min. They took place online via Zoom (version 5.15.11). Between January 2024 and July 2024, the first author conducted interviews with 12 APNs from Switzerland. The interviews lasted between 31 and 60 min. They took place online via Zoom. The questions and topics for the interviews were discussed by the research team and were formulated to cover the roles and competencies of APNs within APN-led clinics, as described in the APN model presented by Hamric and Hanson (2022) [55]. In addition, the questions were formulated with a focus on the specialized area of nursing care for young and middle-aged adults with multimorbidity and complex chronic conditions. Using the internationally recognized APN framework developed by Hamric and Hanson [55] and considering the role of APNs within APN-led clinics, we aimed to identify the key aspects that should be taken into account when developing an APN role in the Swiss context. The interview guide was developed by the research team, informed by the study’s research questions, and internally reviewed and reflected upon to ensure the completeness and clarity of the content. It was based on an initial pool of 20 questions structured into six thematic areas: role perception; competencies and scope of practice; role development and implementation; interprofessional collaboration; the needs and requirements of multimorbid and complex chronically ill adults in younger and middle adulthood; legal frameworks and the current status of APN role development in the United States and Canada. During the revision process, four questions were removed, and six were reworded to improve their clarity and relevance. The final interview guide comprised 16 questions covering the previously defined thematic areas. The order of the questions was flexible so that the interviewer could respond to the individual dynamics of each interview. No external pre-test was conducted. The initial question was as follows: “In what APN role are you involved in the care of multimorbid and complex chronically ill adults of younger and middle age?” Further questions related to various aspects of the role as an APN within APN-led clinics. The interview guide is provided in the Supplementary Materials. At the end of the interview, we collected the participants’ socio-demographic data using a standardized questionnaire. We recorded the interviews digitally and transcribed them word for word. During data collection, an open and exploratory approach was prioritized. Consequently, open-ended questions were employed to elicit participants’ narratives of their experiences with multimorbid and complex chronically ill young and middle-aged adults. Data collection was conducted until code saturation was reached, defined as the point at which no new codes were identified. In addition, meaning saturation was assessed, indicating that no new nuances of meaning emerged within the existing themes. After the sixteenth interview, no new codes were generated; however, three additional interviews were conducted to further confirm the robustness of the findings. Data collection was complete after a total of 19 interviews.

### 2.4. Data Analysis

The interviews were analyzed through the reflexive thematic analysis approach presented by Braun and Clarke (2022) [51]. The process followed a six-step framework [51], implemented in accordance with the qualitative analysis procedure: (1) The transcripts were meticulously reviewed multiple times to ensure thorough familiarity with the dataset. (2) The interviews were systematically coded, with key phrases and sentences being highlighted line by line. At the conclusion of this phase, the corresponding codes were consolidated, resulting in the creation of initial codes. The codes were subsequently compared, and any discrepancies were deliberated and resolved. (3) The compiled list of codes was structured into potential themes and patterns, which were subsequently visualized through a mind map. (4) A research team comprising eight members—three authors (G.B., B.S., and H.M.) and scientists who were not involved in the processes of data collection and analysis—held a detailed discussion to refine and validate the emerging themes and patterns derived from the mind map. (5) These themes were systematically reorganized and critically assessed by revisiting the original quotations. Afterward, the themes were distinctly defined and labeled. (6) MAXQDA 24 (version 26.3) was used to organize the data for the final comprehensive analysis. In the data analysis, an inductive approach was adopted, where codes and categories were derived directly from the data rather than being deduced from a theoretical framework. The evaluation process was deeply reflective, with the first author actively engaging in the research discourse through peer debriefing. This process was particularly valuable during data analysis, enabling careful reflection on the first author’s position.

### 2.5. Rigor

This study adhered to key quality criteria for qualitative research: credibility, dependability, transferability, and confirmability [56]. Credibility was ensured by drawing on the researchers’ personal experience in both academic research and professional practice, as well as by consulting individuals outside the study to identify any issues that may have been overlooked. In addition, all 19 transcripts were coded independently by two researchers to increase their credibility. To ensure transferability, detailed information was provided about the participants, the research environment, and the recruitment process. Confirmability was enhanced by the use of verbatim transcripts and the involvement of individuals not directly involved in the study. Overall, rigor and trustworthiness were ensured through this careful, ongoing process of peer debriefing.

### 2.6. Ethical Considerations

Participants were informed in writing and verbally about the aim, purpose, content, and scope of the study. Each participant signed a consent form and was aware that participation was voluntary and that they could withdraw from the study at any time without consequences. The interviews were transcribed and anonymized, and once the study is complete, the recordings will be deleted in accordance with the cantonal requirements of the ethics committee. The Ethics Committee of Northwest and Central Switzerland (EKNZ) was consulted regarding the necessity of ethical approval for this research project. Subsequently, the EKNZ issued Clarifications of Responsibility (Req-2023-01247, Req-2024-00093), confirming that the project does not fall under the scope of the Human Research Act (HRA), as it does not involve research on human diseases or the structure and function of the human body (HRA Art. 2 para. 1). The investigation conformed to the principles outlined in the Declaration of Helsinki. The participants were informed both in writing and orally about the purpose and scope of the study. Each participant provided written informed consent, acknowledging their voluntary participation and their right to withdraw at any time without consequences.

## 3. Results

Seven APNs from the USA and Canada and twelve APNs from Switzerland participated in this study. The participants from Switzerland were between 34 and 56 years old. Half of the participants had more than 10 years of professional experience in the care of adults with multimorbid and complex chronic illnesses, and the other half had between 5 and 10 years of experience. The specific professional titles of the participants from Switzerland were as follows: APNs (seven), NPs (three), CNS (one), and mixed NP/CNS (one). The participants from the USA were between 38 and 74 years old. Three of the participants had more than 35 years of professional experience, and four participants had between 15 and 20 years of experience in the care of the chosen patient group. The professional titles of the APNs from the USA and Canada were as follows: APNs (three) and NPs (four). Further sociodemographic data of the participants are presented in Table 1 and Table 2.

**Figure 1:**
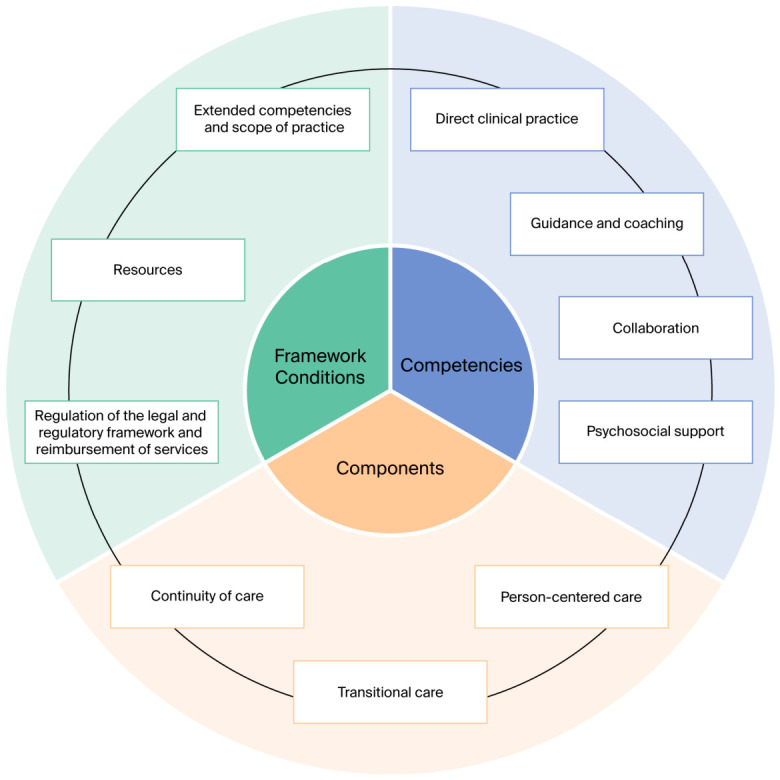
Competencies, components and framework conditions of APNs for the care of multimorbid and complex chronically ill young and middle-aged adults within an APN-led clinic.

The analysis identified a total of 10 themes describing the necessary (1) competencies, (2) components, and (3) framework conditions for the care of multimorbid and complex chronically ill younger and middle-aged adults within an APN-led clinic in a hospital setting in the Swiss context: (1) direct clinical practice, (2) guidance and coaching, (3) collaboration, (4) psychosocial support, (5) person-centered care, (6) transitional care, (7) continuity of care, (8) regulations of the legal and regulatory framework and reimbursement of services, (9) resources, and (10) extended competencies and SOP. A representation of these themes can be found in Figure 1.

### 3.1. Competencies

From the participants’ perspective, the development of an APN role concept for the care of younger and middle-aged adults with multimorbidity and complex chronic diseases within an APN-led clinic in a hospital setting requires the following competencies: (A) direct clinical practice, (B) guidance and coaching, (C) collaboration, and (D) psychosocial support.

#### 3.1.1. Direct Clinical Practice

Direct clinical practice has been emphasized as a necessary core competency of APNs in APN-led clinics for the care of multimorbid and complex chronically ill young and middle-aged adults. A comprehensive clinical assessment was described as a central component of direct clinical practice. This involves a multi-layered approach that includes initial screening and a comprehensive clinical assessments. In addition, a comprehensive needs assessment and a psychosocial assessment should be conducted to capture the complex needs of patients in a holistic manner, accounting for health literacy, readiness for change, and current mental state. Within this process, an evidence-based and person-centred nursing approach is applied to structure care delivery. Further key components include medication management, diagnostic reasoning, and the interpretation of diagnostic tests. Overall, this approach aims to achieve a holistic understanding of patients’ complex care needs:

*“We have a very comprehensive assessment that’s different than what normally happens in the hospital. It’s really to look at high risk situations to pull out those high risks’ potentials. So it could be that there’s underlying anxiety or and depression, or that the there’s not a good support system at home for the patient. We have a whole list of assessments (…) I perform them and then maybe identify. (…) this patient has some underlying anxiety and since they have COPD (…). That is if they become anxious. Is it gonna trigger a COPD flare or if they have a COPD flares, are they going to get more anxious, which is going to make the COPD worse and they end up back in the hospital.”*

(Interview 4, APN, USA, Pos. 16).

From this perspective, within direct clinical practice, getting to know the patient in depth is considered essential; this involves building an understanding of the extent and severity of their conditions as well as their individual risk profile. Particular emphasis is placed on identifying patients’ strengths and resources, alongside their challenges in managing their complex health conditions. Furthermore, patients’ capacity and willingness to adapt to therapeutic interventions is seen as an important aspect of this clinical work:

*“Direct clinical practice is critically important because in your direct clinical practice role you will learn about the person. You will learn about the scope and the extent of their illness, what their what their risks are. You’ll learn enough about them to know what their strengths are, what their challenges are, and how they’re adapting to medical therapies (…) So, I think that is truly foundational.”*

(Interview 1, APN, USA, Pos. 57).

A holistic, evidence-based approach and person-centred care were described as essential in aligning treatments with patients’ individual goals, enhancing patient engagement and building a strong therapeutic relationship:

*“One of the most important tasks is patient engagement. This involves gaining their trust and convincing them that they need to change certain behaviors and make a real effort. Some people simply don’t want to quit smoking, change their diet, or do things we know they should. This kind of engagement is therefore crucial. The APN builds a trusting relationship and tries to motivate the patient.”*

(Interview 1, APN, USA, Pos. 25).

The advanced skills of APNs are expected to have a positive impact on treatment outcomes and to support and monitor patients’ self-management abilities, thereby assisting patients in achieving their goals.

#### 3.1.2. Guidance and Coaching

Guidance and coaching were identified as core competencies of APNs in the care of younger and middle-aged adults with multimorbidity and complex chronic illnesses. These competencies include counseling sessions that provide patients with information, knowledge, and guidance on a range of relevant topics, including partnership, sexuality, fertility, the desire to have children, and how to discuss the severity of the illness with one’s partner and children. The key areas addressed in these sessions include the course of the illness, its progression, and expected outcomes, as well as treatment options and their potential side effects. In addition, counseling covers broader aspects of daily life, including the challenges patients may face in managing their condition, e.g., returning to everyday routines and work and establishing a sense of normalcy in daily life:

*“What undesirable side effects can be expected and how can I prepare them for this, what is possible in terms of their ability to work, and it always depends on whether they are able to do so, or if they are in school or university, what is reasonable and what is possible during the time they are on sick leave, if it is not conceivable or reasonable for them. (…) There are so many possible topics, let’s say, questions or concerns, needs that the person affected comes up with.”*

(Interview 11, APN, Switzerland, Pos. 15–16).

Counseling sessions support patients in promoting their individual autonomy and achieving their personal goals. In addition, counseling sessions empower patients to manage disease-specific symptoms themselves and respond to symptoms and health risks, improving patients’ skills, competencies, and knowledge so that they can integrate their complex chronic conditions, recovery, and the achievement of personal goals into their lifestyle. These sessions also involve motivating and actively engaging patients to manage their complex care needs, as well as providing education, instruction, and coaching to manage complex care needs in relation to health promotion and disease exacerbation. In addition, these sessions aim to support treatment adherence:

*“In these counselling sessions, the focus is on symptom management, self-management, medication management, but also on everyday life (…) it’s about understanding the disease as a whole. Then we have counselling hepatocellular carcinoma counselling and transjugular intrahepatic portosystemic shunt counseling, which are very topic-centred discussions and, of course, cover other issues as well. So, all these social issues, financial issues, everything that actually comes up.”*

(Interview 8, APN, Switzerland, Pos. 11).

These coaching and counseling sessions should also serve a psychoeducational purpose, helping patients to understand their current condition and to cope better with their illnesses. Furthermore, these counseling and coaching sessions are designed to actively involve patients in their treatment, serving as motivation for life changes and supporting patients in their willingness to implement these changes:

*“One of the most important tasks is the active involvement of the patient. It’s about gaining their trust and convincing them that they need to change certain behaviors and truly empower themselves. Some people simply don’t want to quit smoking or change their diet, or they don’t want to do things that we know they should be doing. Therefore, this form of involvement is important, and the nurse builds this trusting relationship and tries to motivate the patients. (…) You have to find out what will motivate this patient to approach things differently (…) what is important to him. I think in healthcare we rely too much on patients doing something simply because we as healthcare providers consider it important—and that is not the truth. You have to ask yourself: Why is this important to the patient?”*

(Interview 1, USA, Pos. 25–49).

#### 3.1.3. Collaboration

Collaboration was identified as an essential core competency for Advanced Practice Nurses (APNs) in the care of this patient population. APNs are required to collaborate at multiple levels within reciprocal professional relationships and—with a view to the entire treatment process—to coordinate the exchange and communication between various healthcare professionals and departments. This includes collaboration between APNs, their patients, and their families to develop a treatment plan, improve self-management of complex needs and behaviors, optimize symptom management for chronic conditions, and optimize medication use and lifestyle changes. It also includes collaboration with interprofessional teams of physicians, nurses, physical therapists, and nutritionists within the hospital who are involved in the patient’s care, with the aim of implementing an evidence-based treatment plan and coordinating discharge. Finally, it includes collaboration and continuous communication with the external healthcare professionals involved in a patient’s care, such as general practitioners, clinicians, specialists, physical and occupational therapists, nutritionists, and psychologists. In addition to communication skills, collaboration requires strong interpersonal competencies such as empathy, flexibility, responsibility, trust, problem-solving skills, the ability to motivate, coordination skills, and leadership skills. APNs must be able to understand the perspectives of all parties involved and adapt the care process accordingly. In doing so, they integrate and utilize the specific expertise of different healthcare professionals to ensure and coordinate the best possible patient care:

*“I have a patient; he’s 58 years old and has multiple sclerosis. In addition, he suffers from chronic nausea and vomiting, neurogenic bladder, kidney stones, and recurrent urinary tract infections. And then there’s the gout. He was in the Marine Corps (…). He used to be a very fit, athletic person, but now he’s in a wheelchair (…) So, he’s being treated by six specialists plus a general practitioner, and they’re all working in different directions. A rheumatologist is also involved because of his gout. As you know, medications for gout can cause nausea. Medications for MS can cause urinary tract infections. That’s why we have to find a common approach, for example, to reduce the dose of some medications and thus alleviate the side effects (…) So, it’s the typical situation of a middle-aged man with multiple illnesses. His case is very complex. As I said, I pointed out today that everyone has to pull together instead of each focusing on just one different organ system.”*

(Interview 2, Pos. 59–60).

#### 3.1.4. Psychosocial Support

Providing psychosocial support in the care of younger and middle-aged adults with multiple illnesses and complex chronic diseases was highlighted as an additional essential core competency of APNs. During the acute and post-acute treatment phases, these patients experience a high level of psychosocial distress, accompanied by emotional and psychological distress, depression, and anxiety disorders. In particular, following diagnosis, they require psychosocial support to stabilize their mental state and to cope with the course of the illness and its consequences—both in the hospital during the acute phase and at home after discharge:

*“Absolutely, I mean, that’s part of our job, because the emotional strain is high. People who are diagnosed with cirrhosis have a life expectancy of 12 years. When they experience their first decompensation, i.e., when they are hospitalized due to comorbidities, be it ascites, hepatic encephalopathy or hemorrhaging, they have a life expectancy of 2 years. So, they have a chronic progressive disease that usually leads to premature death.”*

(Interview 8, APN, Switzerland, Pos. 61).

Patients experience a high level of psychosocial and psychological distress due to existential anxieties and fears about the future, such as professional and financial worries, as well as other problems, such as the fear of job loss, unemployment, and disability. In addition, concerns related to family members, including spouses or minor children, contribute to this burden and are often linked to perceived loss of relationships and role changes within the family system, social exclusion, isolation, and loneliness. These concerns necessitate psychosocial support for this patient group and may require referral to psychological or psychiatric services. In this context, APNs must be able to recognize psychosocial stress, emotional distress, and potential anxiety disorders as part of comprehensive patient care; they must also assess whether the distress can be managed independently or whether referral to psychological or psychiatric services is required:

*“As soon as the diagnosis is made, I am present at the diagnostic consultation (…) and always assess the psychosocial needs. Last year, I also completed the Certificate in Advanced Studies (CAS) in psycho-oncology (…) if the level of psychological distress is manageable, I can also help with psycho-oncological counselling or refer them to psycho-oncologists.”*

(Interview 5, APN, Switzerland, Pos. 25).

### 3.2. Components

Several interconnected components were found that, from the perspective of the participating APNs, are necessary for developing an APN role concept for the care of younger and middle-aged adults with multimorbidity and complex chronic conditions in an APN-led clinic within a hospital setting. These components were perceived as closely interconnected; prior to discharge, an understanding of each individual, patient-specific situation is considered to be a key prerequisite for ensuring effective transitional care; in turn, this is seen as essential for maintaining continuity of care throughout the entire treatment process. These components include the following: (E) person-centred care; (F) transitional care; (G) continuity of care.

#### 3.2.1. Person-Centred Care

Person-centred care was highlighted by participants as a central key component for optimizing the care of younger and middle-aged adults with multimorbidity and complex chronic illnesses. This encompasses a holistic view of patients, their illnesses and medical needs, and their personal needs and problems and challenges they face. Especially prior to discharge, it is essential to identify what a patient needs to live independently at home and ensure that these needs are being met in their individual situation. The central focus is on the patient’s goals and supporting them in achieving these goals:

*“It’s very much focused on the patients’ goals, to help them achieve their goals, to learn how to live with and manage and be healthier with their, whatever their health problems are (…) the APN will look at everything related to that goal. (…) So, using that as a motivator, we get all kinds of things in place, we get community resources in place, we get the family engaged. (…) Is the big picture of what’s it going to take to help this person be successful. And many times, it’s to help the patient be able to take care of themselves where before they were not able to do that, but we helped them and through education and through also working with the providers about better managing the patients. Sometimes they need to have you on different drugs or whatever.”*

(Interview 1, APN, USA, Pos. 21–23).

From the perspective of APNs, in addition to building a trusting relationship with patients, their active participation is considered another essential aspect of person-centred care—this comprises shared decision making, patient involvement, and the opportunity to participate in treatments:

*“I think that participation is an important need. They want to be involved in their treatment, i.e., autonomy and participation. It’s not like it used to be (…) when therapy was initiated and patients couldn’t contribute much. Now patients are informed, they know what’s going on, they want to be informed, they want to be involved to treat (…) to involve the patients, and that’s a joint treatment, it’s the joint initiation of therapies with participatory decisions by the patients, and it’s not just that the person with the white coat decides what happens, autonomy is really something that must not be forgotten and is really very important.”*

(Interview 10, APN, Switzerland, Pos. 64).

From the participants’ point of view, an essential aspect of person-centered care is the recognition of each patient’s values and beliefs, as well as the acceptance of their decisions:

*“At the next consultation, they came and said they would now like to draw up a living will with me (…) they were enormously relieved that they had now put it down on paper, and although I am a little surprised by the content, because he really wanted maximum therapy until the end of his life. He said he was a fighter, that he had already been through a lot, that he wanted everything to be done (…). We then recorded the patient’s clear wishes and intentions.”*

(Interview 3, APN, Pos. 51).

#### 3.2.2. Transitional Care

Transitional care was highlighted as an additional key component in the care of multimorbid and complexly chronically ill younger adults during the transition from hospital to home. From the perspective of APNs, transitional care represents a central aspect of their role; here, they provide the support necessary to manage the highly complex care and treatment needs of this patient population in outpatient settings. Transitional care involves comprehensive and holistic care delivered in collaboration with patients, their families, and interprofessional teams. Particular emphasis is placed on individualized, person-centered care and coordination of care, as well as support for self-management, symptom control, and medication adherence. In addition, transitional care includes assistance with psychosocial concerns and the organization of daily life:

*“Transitional care means that if a person is going from one clinical care setting to home that they come into the hospital, they were completely healthy, they had a heart attack and now they’re going home very, very ill. So, they’re not just coping with the change insight, they’re coping with a major difference in their level of function. So, it’s not just setting specific it’s also what, what, the needs are of the patient. And what we have found over years is if you don’t manage those transitions, people get into a lot of trouble and they, even though sometimes in the hospital, we think that we’re preparing patients well to go home many times we don’t really know what their issues are because we haven’t been in the home. We don’t know what happened. We don’t know what the support systems are.”*

(Interview 1, APN, USA, Pos. 21).

Another important aspect from the perspectives of APNs in the context of transitional care—coordinating the transition from hospital to home—is ongoing support, including prioritizing appointments to reduce patients’ feelings of being overwhelmed, especially after discharge following an acute episode:

*“Coordinating appointments with the general practitioner or cardiologist, as I often find that patients are simply overwhelmed when they are at home, they can’t keep up with everything they still have to do and often don’t even know what that is anymore (…)”*

(Interview 6, APN, Switzerland, Pos. 21–23).

#### 3.2.3. Continuity of Care

Continuity of care emerged as another key component in the care of these patients. From the perspective of APNs, continuity of care ensures seamless care from hospital admission to discharge and beyond, delivered through the coordinated involvement of the same APN in collaboration with patients, their families, and internal and external healthcare providers. Continuity of care primarily serves to prevent interruptions in care across different healthcare settings during the transition from hospital to home. This is facilitated by an APN, who coordinates and delivers the necessary interventions throughout the entire course of treatment. In this context, continuity of care for patients with complex conditions strengthens communication and collaboration between hospital-based teams and outpatient healthcare providers. APNs emphasize that it is necessary that they take on a central coordinating role throughout the entire treatment process, both within and between different care settings, in order to ensure continuity and coordination of care, particularly for patients with complex conditions. This role includes building a therapeutic relationship with patients, gaining their trust, and actively involving them in their care:

*“During the hospital stay, I see the patients daily and work closely with the attending physician, the nursing staff, the social workers, the discharge planners, and all the outpatient services involved after the patient’s discharge. I’m like the quarterback in the football—I coordinate the various players and ensure everyone stays informed about their respective tasks (…) That’s my role in the hospital: I help determine the appropriate time for the patient’s discharge, identify the necessary discharge services, and support the social workers and discharge planners in this. I offer home visits and work as part of a team and so we can manage it together.”*

(Interview 4, APN, USA, Pos. 16–17).

APNs describe themselves as the first point of contact not only for patients and their families but also for rehabilitation services and other healthcare professionals, including general practitioners, specialists, physiotherapists, and speech and language therapists. From the perspective of APNs, they are also responsible for referring patients to new healthcare services and monitoring their treatment progress to ensure that these complex cases receive the necessary high-quality care in a timely manner. APNs consider it important to revisit topics and information already discussed during hospitalization when conducting home visits in order to clarify outstanding questions and support patients in accessing and utilizing external support services. Given the complexity of care and the high level of psychosocial distress in this patient population, continuity of care and APN support represent a key responsibility in the management of complex, chronic, and progressive conditions in this patient group. In this context, APNs, in collaboration with primary care physicians, support young and middle-aged adults with multimorbid and complex chronic conditions in transitioning from intensive transitional care programs to independent management.

### 3.3. Framework Conditions

Several interdependent framework conditions were identified in the perspectives of Advanced Practice Nurses (APNs) as necessary prerequisites for the development of an APN role concept for the care of younger and middle-aged adults with multimorbidity and complex chronic diseases in an APN-led clinic within a clinical setting. These are the following: (H) regulation of the legal and regulatory framework and reimbursement of services; (I) resources; (J) extended competencies and scope of practice.

#### 3.3.1. Regulation of the Legal and Regulatory Framework and Reimbursement of Services

APNs emphasized the existence of an appropriate legal and regulatory framework as a key factor influencing the feasibility of practicing as an APN in an APN-led clinic for multimorbid and complexly chronically ill adults of younger and middle age. Participants described the current legal and regulatory framework for APNs in Switzerland as insufficiently developed, resulting in uncertainties regarding formal recognition of the APN role, its scope of practice, and its professional responsibilities. They highlighted the need for a clear legal definition of the APN role, including the regulation of scope of practice and requirements for professional practice. Furthermore, participants referred to the importance of standardized regulations governing licensure, credential recognition, and professional oversight. The legal recognition and regulation of APN master’s degree programs were also considered essential for ensuring consistent educational standards and strengthening the professional foundation of the APN role:

*“It would be great if the Master’s degree were also legally regulated and equipped with expanded competencies (…). It’s simply unacceptable that we still haven’t clearly defined and formulated these roles. And that there’s still a lack of clarity between the individual APN roles—I’m talking about CNS and NP—we still have many hybrid forms here in Switzerland because we don’t have the training for CNS and NP at the level one would expect. It’s actually a tragedy when you consider that we’ve been able to study in this field for 24 years now, but nothing has changed at the national level.”*

(Interview 8, APN, Switzerland, Pos. 69).

The participants emphasized that, in addition to a clear legal and regulatory framework, the reimbursement of services provided by APNs should also be regulated by law. The ability to independently bill for services was identified as an important prerequisite for working in APN-led clinics.

*“It would also be a great simplification if tariff structures were such that APNs could also clearly bill 1:1 for the services they could provide and do provide. A lot of things are now in a grey area where the doctor is billed, under his name, even though the service is provided by me.”*

(Interview 11, APN, Switzerland, Pos. 71).

#### 3.3.2. Resources

The availability of resources to fulfil the APN role in the care of younger and middle-aged adults with multimorbidity and complex chronic illnesses was emphasized as another important aspect. Participants noted that vacant positions and unfilled posts represent a significant challenge. Ensuring the sustainability of the APN role was highlighted as a key concern, alongside the integration of APNs as an integral part of the multidisciplinary care team, beyond the acute setting and across the outpatient sector:

*“A major issue with APNs is that they are ’single-man, single woman roles’ and that APN teams are rarely available, where patients have the option of having a contact person available in times of absence. This is not the case in my role either (…) Ensuring the sustainability of the role is another issue (…) if I were to reorient myself, would the sustainability of the role be ensured, that the role and the position would be filled again, even if one is aware of the added value (…) APN roles should be seen as an integral part of the treatment team, even beyond the acute setting and across the outpatient sector.”*

(Interview 11, APN, Switzerland, Pos. 71).

Participants emphasized that funding for APN roles should be ensured at the national level in Switzerland. In addition, they highlighted the need to develop mechanisms to demonstrate the effectiveness of the APN role in the care of this patient population:

*“I secured funding through an innovation grant application, and we received enough money to fund one role for a year (…) it’s difficult to clearly demonstrate the effectiveness of such roles, and with the limited scope of practice we have in Switzerland due to the legal situation, it’s even more difficult because we can’t easily conduct these comparative studies with APN physicians (…) you can’t score points with patient or team satisfaction.”*

(Interview 8, APN, Switzerland, Pos. 39).

The participants from Switzerland perceived a lack of full-time positions as a key resource-related limitation in attempts to optimize and improve the care of young and middle-aged adult patients with complex needs and multimorbid, chronic illnesses. This shortage was seen as a limiting factor in the continuity of care that APNs are able to provide, given the restricted scope of their role. Furthermore, insufficient time resources for patient follow-up were identified as a factor constraining systematic assessments and management of patients’ needs and requirements:

*“I would need much more time for follow-up care (…), but I also see from what patients say that it’s not just a case of everything being fine when the illness is under control (…) assessing needs in the follow-up phase (…) that would need to be expanded.”*

(Interview 5, APN, Switzerland, Pos. 133).

#### 3.3.3. Extended Competencies and Scope of Practice

Participants identified a need for extended competencies and a clearly defined SOP for APNs within APN-led clinics to better address the complexity of care for young and middle-aged adults with multimorbidity and complex chronic conditions. This included, on the one hand, the authority to manage medications, including prescribing and adjusting treatment regimens; on the other hand, participants reflected that the authority to refer patients to external healthcare professionals would be beneficial in their ability to support the continuation of complex outpatient therapies for this patient population. The participants from the USA and Canada stated that APNs have the necessary skills to independently involve the necessary supporting health professionals such as physiotherapists, nutrition therapists, and psychologists; they also asserted their knowledge of the services that could ensure and facilitate the necessary continuity of care for these multimorbid and complex chronically ill younger and middle-aged adults, both within the hospital and after their hospital stay. To acquire these necessary advanced skills, and to gain an understanding of the role of APNs within APN-led clinics in relation to transitional care, a comprehensive training program with online modules was developed to acquire the necessary knowledge:

*“We therefore have an entire educational program we’ve developed we have online modules course modules. We have a whole course that we do on transitional care, that the nurses can do online to really learn all of this because they tend to have been educated in the model of the role that you’re in, where you have your patient in clinic and you follow that patient, but you don’t have that broad experience across the full spectrum of healthcare. So, we need to help them understand.”*

(Interview 1, APN, USA, Pos. 26).

The independent adjustment of medication—particularly for patients with heart failure and especially in the early post-discharge phase—was identified as an area requiring extended competencies for APNs. Participants indicated that this field of activity should be reviewed and adapted at the legislative level to enable earlier and more effective treatment adjustments. This was seen as a means of improving patient outcomes by facilitating the timely optimization of therapy:

*“The adjustment of medication in the early phase of discharge home is an essential aspect of ‘offering an additional service so that patients can be more stable (…) this includes, for example, titrating medication, or if we let them (the patients) run on half the dosage, then we are simply giving them the opportunity to have a better outcome.”*

(Interview 3, APN, Switzerland, Pos. 35).

Given the absence of a fully established legal and regulatory framework for APNs in Switzerland, participants highlighted the need for clear legal regulations as well as structured educational programs to support the expansion of competencies and SOP. They indicated that, in the context of a limited SOP, a so-called “collaboration practice physician” would be required; this would be similar to models implemented in certain states in the USA such as Pennsylvania, where collaborative practice agreements are used.

## 4. Discussion

The results of this study highlight the competencies, components, and framework conditions that are necessary prerequisites in the development of an APN role in an APN- led clinic in Switzerland for multimorbid and complex chronically ill young and middle-aged adults within clinical setting in the Swiss context. The required competencies identified for this group are direct clinical practice, guidance and coaching, collaboration, and psychosocial support; the required components identified for this group are person-centered care, continuity of care, and transitional care; the essential framework conditions identified for this group are the regulation and regulatory of the legal framework and the eligibility for reimbursement of services, resources, and extended competencies and scope of practice.

The description of direct clinical practice as a core APN competency aligns with the framework presented by Hamric and Hanson (2022) [57], who define it as a central element of APN practice. The authors emphasize a holistic approach that accounts for both physical and emotional patient needs to support stabilization and the improvement of health outcomes [57]. Similarly, guidance and coaching are key responsibilities in current APN roles, consistent with frameworks such as the International Council of Nurses (2020) [44] and that presented by Hamric and Hanson (2022) [55]. As relationship-oriented approaches, guidance and coaching provide information and support to younger adults with multimorbidity and complex chronic illnesses, promoting self-determination, autonomy, goal setting and achievement, and coping with changes in their lives [57]. Guidance and coaching are considered key responsibilities, involving education and support to help patients in managing complex health needs, promoting adherence, and developing the skills needed for self-management of symptoms and chronic conditions. Equally, collaboration has been identified as another key APN competency, occurring across multiple levels through reciprocal relationships. The identification of this competency aligns with the findings of Hamric and Hanson (2022), who describe collaboration as a core APN competency, involving reciprocal relationships with patients, their families, and healthcare providers across the treatment process [57]. According to Hamric and Hanson, this collaboration includes coordinating communication across the treatment process, considering patient preferences, and using interprofessional resources to ensure high-quality care. The ability and competence to provide psychosocial support for this patient group has been confirmed as another essential ANP competency, both in the hospital during acute episodes and at home after discharge.

This significant psychosocial burden experienced by multimorbid and complexly chronically ill younger and middle-aged adults is characterized by higher levels of psychological distress [17], mental disorders [14,22,23], impaired work performance and productivity [6], early retirement [58], reduced social contacts to cope with treatment burden and meeting family needs [59,60], and negative effects on family relationships [58,61,62]. In Hamric and Hanson’s (2022) competency model, psychosocial support is embedded within several competency areas, such as direct clinical practice and guidance and coaching, but is not identified as a standalone competency [57]. Given the high psychosocial burden in this patient group, psychosocial support should be recognized as a distinct APN competency. This would better reflect the growing importance of psychosocial aspects in the care they provide.

Another key finding of this study is that the care of young and middle-aged adults with multimorbidity and complex chronic illnesses provided by APNs should be person-centered. This involves a holistic view of patients, their illnesses and medical needs, as well as their individual challenges and concerns. Recognizing and addressing each patient’s specific needs is essential in supporting their ability to live independently at home. In addition to building a trusting relationship with the patient, this includes promoting their participation through shared decision making, patient involvement, and active engagement in their treatment. The focus of person-centered care is not only on physical and cognitive outcomes but also aims to enhance the wellbeing of those affected, recognizing their authenticity and seeing the person behind the illness [63]. Person-centered care also considers the perspectives of Swiss APNs themselves, actively shaping their work environment and thus further developing nursing practices [63]; this involves implementing improvements which they identify as being necessary for the care of this patient population (these are improvements that APNs in the US and Canada have identified as being important). This person-centered view of multimorbidity differs from the disease-centered comorbidity perspective of previous care models [64] and highlights the paradigm shift from a disease-specific biomedical approach to a holistic, biopsychosocial understanding of health and care for multimorbid and complexly chronically ill younger and middle-aged individuals [65]. This approach ensures that patients with multiple chronic illnesses and functional limitations have unique, individual experiences that go far beyond the sum of their separate diagnoses. The associated complexity of health and treatment burdens requires a comprehensive, holistic, person-centered approach that systematically considers and integrates the individual needs, personal experiences, and values of this patient group, going beyond disease-specific care strategies [66]. However, it is crucial to clearly define the future focus and goal of APN roles in the care of multimorbid and complexly chronically ill young and middle-aged adults in Switzerland. This is important: if the focus of APN roles is on supporting and replacing physicians, rather than on person-centered, health-oriented, and holistic nursing practice, the nursing components of the APN role will be undervalued and neglected [67]. This tendency is reinforced in disease-oriented and medically driven healthcare systems, which often fail to adequately recognize the value-adding components of the APN role. Furthermore, there is currently a lack of reimbursement mechanisms that appropriately recognize and reward these broader dimensions of care [68].

This study also indicates that the experiences of APNs from the USA and Canada in the care of this population group in clinical settings are strongly linked to the concept of transitional care; these individuals consider transitional care to be a central focus of their work. Transitional care includes elements of comprehensive, holistic care in collaboration with patients and an interprofessional team. Transitional care plays an exceptionally important role, particularly for adults with complex chronic illnesses during the transition from hospital to home; this has been shown by studies demonstrating its effectiveness in ensuring continuity of care, preventing clinical deterioration, and supporting safe transitions between hospital and home [69,70,71]. Transitional care models are internationally recognized as the most innovative care models for ensuring the continuity of care from hospital admission to discharge. They also demonstrably contribute to improved patient safety and reduced costs [72,73]. However, in this study, the experiences of Swiss APNs demonstrate that this transitional care component, which is needed by this patient population, is still underdeveloped, and it is less mature than in the USA and Canada. The acquisition of knowledge and skills for managing transitions of care, particularly among adults with complex care needs, should therefore be an integral component of APN education. Educational programs could follow the example of the United States, where a competency-based approach has been developed to prepare future APNs for the care of older adults. This approach is grounded in the evidence-based interventions of the transitional care model and aims to strengthen competencies for delivering high-quality, coordinated transitional care [74].

Another finding of this study is the identification of several interdependent framework conditions as necessary prerequisites for developing an APN role concept for the care of younger and middle-aged adults with multimorbidity and complex chronic diseases in an APN-led clinic within a hospital setting. These necessary prerequisites include the availability of essential resources such as time and personnel, the regulation of legal and regulatory frameworks, and the reimbursement of services provided by APNs in Switzerland. The resource debate represents a crucial issue for Swiss APNs in ensuring the continuity of care for these patients with complex needs and the assessment and fulfillment of their needs. Within Switzerland, CNS roles still predominate. Due to the Swiss financing system, these CNS roles can be integrated into clinical settings using diagnosis-related groups (DRGs). The deployment of Swiss APNs as NPs, primarily in the outpatient setting, is different. They are not yet authorized as independent service providers under the Swiss Health Insurance Act. An important step would be the formal recognition of APNs within the Swiss Health Professions Act in order to advance this issue at the national level [75]. Initial developments in Switzerland indicate that the APN role is being regulated at the cantonal level, particularly in the cantons of Vaud, Neuchâtel, and Valais [75]. Initial positive experiences in these new APN roles have been documented [76,77,78,79,80,81,82,83,84]. Likewise, the development of a legal framework for Swiss APNs should be accompanied by an expansion of their competencies, enabling them to independently refer patients to internal and external healthcare professionals such as physiotherapists, nutritionists, and psychologists. This may also include expanding their competencies to prescribing and adjusting medications. However, the further development of APNs in Switzerland must be understood within a highly regulated healthcare system in which workforce planning, financing mechanisms, and professional scopes of practice are closely interlinked. Unlike in less-regulated systems, the integration of APNs requires coordinated developments across multiple policy domains, including education, professional regulation under the Health Professions Act, and reimbursement structures within the mandatory health insurance system. From a workforce perspective, the introduction of APNs raises critical questions regarding role delineation between physicians and advanced nursing roles, as well as the redistribution of clinical responsibilities in response to increasing care complexity and workforce shortages. In terms of financing, a key barrier remains: the current absence of clearly defined APN reimbursement pathways. This necessitates the development of tariff structures and potential new categories of ambulatory service provision. These regulatory and financial conditions directly influence educational requirements, reinforcing the need for competency-based master’s-level preparation that integrates advanced clinical training, supervised practice, and alignment with internationally established APN standards. Consequently, the further development of APNs in Switzerland is not a singular regulatory step; rather, it is a multi-layered policy process requiring alignments between education, workforce regulation, service authorization, and healthcare financing. It is therefore essential that these aspects are appropriately regulated and coherently addressed to ensure the sustainable integration of APNs into the Swiss healthcare system [75] (p. 26–38). The article by Hako, Turunen, and Jokiniemi (2023) [85] demonstrates that clearly defining competencies for Advanced Practice Nurses (APNs) is crucial for the successful development and implementation of this professional role in healthcare. Since competence is a fundamental prerequisite for APN practice, identifying and defining their competency dimensions forms the basis for developing role profiles, training programs, and competency assessment procedures. Furthermore, systematically documenting APN competencies helps strengthen role understanding, increase the visibility of the professional role, and support its implementation in healthcare. This, in turn, can improve the quality of care, access to healthcare, and the cost-effectiveness of healthcare professional deployment [85].

Overall, APN-led care models demonstrate positive effects on clinical and patient outcomes in the care of multimorbid and complex chronically ill adults of young and middle age, meanwhile, high-intensity integrated care approaches do not consistently lead to cost reductions [46]. The current evidence suggests that integrated healthcare is primarily focused on geriatric populations [38,86,87], whereas younger and middle-aged adults remain underrepresented [46]. At the same time, there is considerable heterogeneity among APN-led care models, including transitional care models, care management models, care transition intervention programs, nurse case management, and chronic disease management models. This heterogeneity is also reflected in the competencies of APNs, which vary in both form and intensity. Furthermore, these models differ substantially in duration, as well as in the average number and intensity of patient contacts [46]. From a research perspective, this implies that a logical next step in the development of APN roles could involve the systematic design and further development of a new APN role concept using the PEPPA-Plus framework, which would subsequently be adapted, implemented, and evaluated within the respective institutional context. This process could be undertaken as part of a feasibility study [88]. In this context, it would also be essential to systematically capture additional perspectives on the APN role, particularly those of external healthcare providers, such as general practitioners and community-based home care services. The implementation of the APN role concept should be guided by a program theory, consisting of a change model and an action model [89]. This program theory later supports a theory-driven evaluation, as recommended by the latest guidelines of the Medical Research Council (MRC) Framework for Developing and Evaluating Complex Interventions [90], which emphasizes a responsibility “not only to assess whether an intervention works or does not work but also how and why it does work” [89] (p. 17). This evaluation could also serve as a basis for decision making regarding the implementation of an intervention in collaboration with policymakers [91].

## 5. Conclusions

This is the first study to identify the necessary competencies, components, and framework conditions for developing an APN role concept for the care of younger and middle-aged adults with multimorbid and complex chronic illnesses in Switzerland. The perspectives of APNs in Switzerland show that this patient group needs complex and long-term care that extends beyond the boundaries of the hospital. While the development of APN roles in Switzerland is still in its early stages, the findings suggest that Swiss APNs may be well positioned to contribute to this role.

In the context of these findings, this section presents key conclusions and recommendations for policy development, the further advancement of APN education, and future research within the Swiss context.

Education and continuing professional development remain central to fully unlocking the potential of APNs in Switzerland. In particular, the further development and harmonization of existing Master of Science (MSc) programs in nursing are crucial to ensuring consistent competency profiles across the country and to strengthening the APN role in a coherent manner. In addition, a clear embedding of the APN role within the Health Professions Act is necessary to clearly define educational requirements, competencies, responsibilities, and scope of practice. Only in this way can the coherent integration of APNs into the Swiss healthcare system be ensured.

The findings highlight the importance of expanding the competencies and scope of practice of APNs in Switzerland in the care of multimorbid and chronically ill young and middle-aged patients with complex healthcare needs. A clear nationwide legal framework is necessary to enable APNs to take on more autonomous roles in the management of complex patient populations and thereby contribute to the sustainability of the healthcare system. Experiences from countries with established APN roles, such as the United States and Canada, further demonstrate the benefits of integrating APNs into APN-led care models within hospital settings, including improved patient outcomes and healthcare utilization outcomes such as reduced hospital readmission rates and shorter lengths of stay.

Future research should focus on the implementation and outcomes of APN-led models of care in hospital settings, including evaluations of patient outcomes, readmission rates, and quality of care, as well as the long-term sustainability of such models. In addition, further studies are needed to examine the development of APN roles over time in Switzerland and to identify factors that might facilitate or hinder their successful implementation. Moreover, future research should explore the perspectives and experiences of healthcare professionals and patients regarding APN-led care models in hospital settings. Funding for projects promoting the APN role is essential in supporting care for young and middle-aged adults with multimorbidity and complex chronic conditions.

## 6. Limitations

A key limitation of this study lies in the fundamental differences between the healthcare systems included. The study utilizes perspectives from APNs in the United States and Canada, where APN roles within APN-led models of care for multimorbid and complex chronically ill adults in younger and middle adulthood are well established; these are combined with perspectives from Switzerland, where APNs are present but APN-led models of care for this patient group have not yet been implemented. This structural difference represents a fundamental contextual discrepancy between the settings and perspectives accounted for in this study. While the integration of these perspectives enables a broader understanding of the APN role in the care of multimorbid and complex chronically ill younger and middle-aged adults, this tension cannot be fully resolved and therefore remains a limitation of the study.

## Figures and Tables

**Table 1:** Sociodemographic data of APNs from Switzerland.

	APNs (*n* = 12)
Age (year)	
Mean Age	42
Range (SD)	32–56 (8.2)
Sex	
Female	10
Male	2
Institution	
Medical Center	1
City Hospital	4
University Hospital	7
University degree	
Master of Science in Nursing	10
PhD in Nursing Science	2
Years of work experience	
30–40 years	3
20–29 years	3
10–19 years	5
<10 years	1
Years of experience in current position	
20–25 years	1
10–19 years	1
5–9 years	3
<5 years	7
Experience working with patients with multimorbidity and complex chronic conditions	
20–30 years	2
10–19 years	4
<10 years	6

Abbreviations: APNs, Advanced Practice Nurses; SD, standard deviation, *n*, number of participants.

**Table 2:** Sociodemographic data of APNs from the USA and Canada.

	APNs (*n* = 7)
Age (year)	
Mean Age	56
Range (SD)	38–74 (11.5)
Sex	
Female	7
Male	0
Institution	
Heart Institute	1
Hospital for Veterans	1
University Hospital	5
University degree	
Master of Science in Nursing	5
PhD in Nursing Science	2
Years of work experience	
>45 years	1
35–40 years	2
20–25 years	3
<20 years	1
Years of experience in current position	
>30 years	1
10–20 years	4
1–5 years	2
Experience working with patients with multimorbidity and complex chronic conditions	
>45 years	1
30–40 years	2
20–30 years	3
>15 years	1

Abbreviations: APNs, Advanced Practice Nurses; SD, standard deviation, *n*, number of participants.

## Data Availability

Data are available upon request in order to guarantee the anonymity of the participants.

## Supplementary Materials

The following supporting information can be downloaded at: https://www.mdpi.com/article/10.3390/healthcare14121779/s1, Table S1: Standards for Reporting Qualitative Research: A Synthesis of Recommendations. Supplementary File S1: Interview guide.

## Abbreviations

The following abbreviations are used in this manuscript:

APN	Advanced Practice Nurse
CNS	Clinical nurse specialist
COPD	Chronic Obstructive Pulmonary Disease
EKNZ	Ethics Committee of Northwest and Central Switzerland
HCC	Hepatocellular carcinoma
HRA	Human Research Act
ICN	International Council of Nurses
MSc	Master of Science
MRC	Medical Research Council
MS	Multiple sclerosis
NP	Nurse practitioner
PEPPA	Participatory, Evidence-based, Patient-focused Process
SOP	Scope of practice
SRQR	Standards for Reporting Qualitative Research
TIPS	Transjugular intrahepatic portosystemic shunt
USA	United States of America
