# Supplementary material for: The Role of Advanced Practice Nurses in the Care of Multimorbid and Complex Chronically Ill Young and Middle-Aged Adults in Hospital Settings—Perspectives on Experience of APNs: A Qualitative Study
Source: Healthcare (Basel). 2026 Jun 19;14(12):1779. doi: 10.3390/healthcare14121779 (PMC13299316; doi:10.3390/healthcare14121779)
Supplement: Supplementary file 1 [file healthcare-14-01779-s001.zip › Supplement_ Interview guide.pdf]

Interview guide:

1. In what APN role are you involved in the care of multimorbid and complex chronically ill adults of younger and middle age?

2. For which patient population do you work in your role?

3. What is your Scope of Practice during the hospitalization, the transition from hospital to home and after hospitalization at home for these patients?

4. What is your scope of practice as an APN for this patient population during the hospital stay, during the transition from hospital to home, and at home?

5. How did the development and implementation of your APN role take place?

6. Which problems have you been confronted with in your role as an APN?

7. What facilitating and hindering factors have you experienced in implementing these APN roles?

8. Who is part of the interprofessional team, and what are your experiences with it?

9. What are the needs and requirements of young and middle-aged adults with multimorbidity and complex chronic conditions in the hospital and at home?

10. What additional activities could further improve patient care?

11. You are familiar with the Hamric model of APN practice and its core competencies - direct clinical practice, guidance and coaching, collaboration, leadership, evidence-based practice, and ethical practice. In your opinion, which of the above-mentioned competencies are required for APNs in an APN-led clinic to care for and treat multimorbid and/or complex chronically ill young and middle-aged adults?

12. Do the above-mentioned competencies sufficiently describe your role?

13. Do APNs need to meet additional requirements that are not described in Hamric's APN model?

14. What additional activities could further improve patient care?

In addition, these two interview questions were asked to participants from the United States and Canada.

15. What is the legal framework of APN practice in the United States/Canada?

16. What is the current status of APN role development in the United States/Canada?
